# Sustained Virologic Suppression After Discontinuation of Long-acting Cabotegravir/Rilpivirine: A Two-case Series Highlighting Unexpected Heterogeneity in Pharmacokinetics and Viral Control

**DOI:** 10.1093/ofid/ofag465

**Published:** 2026-08-11

**Authors:** Annalisa Marinosci, Sabine Yerly, Catia Marzolini, Alexandra Calmy

**Affiliations:** HIV Unit, Division of Infectious Diseases, Geneva University Hospitals (HUG) and University of Geneva, Geneva, Switzerland; Laboratory of Virology, Geneva University Hospitals (HUG), Geneva, Switzerland; Service of Clinical Pharmacology, Department of Laboratory Medicine and Pathology, Lausanne University Hospital and University of Lausanne, Lausanne, Switzerland; Division of Infectious Diseases, Departments of Medicine and Clinical Research, University Hospital Basel and University of Basel, Basel, Switzerland; Centre for Experimental Therapeutics, University of Liverpool, Liverpool, UK; HIV Unit, Division of Infectious Diseases, Geneva University Hospitals (HUG) and University of Geneva, Geneva, Switzerland

**Keywords:** cabotegravir, HIV, long-acting, pharmacokinetic, rilpivirine

## Abstract

Long-acting (LA) injectable cabotegravir (CAB) and rilpivirine (RPV) are recommended for maintenance treatment in virologically suppressed people with HIV-1. A key concern with this strategy is the prolonged pharmacokinetic “tail” after treatment discontinuation, during which declining drug concentrations may increase the risk of viral rebound and resistance selection. Current clinical guidance therefore recommends prompt initiation of alternative antiretroviral therapy if injections are interrupted. We report two people with HIV who remained virologically suppressed (HIV-1 RNA <50 copies/mL) for several months after discontinuation of LA CAB/RPV without any documented alternative antiretroviral therapy. The first case involved a 54-year-old man with well-controlled HIV-1 disease who had received 13 CAB/RPV injections over approximately two years before treatment interruption. After missing scheduled visits, he returned to care seven months after his last injection with persistent viral suppression and plasma drug concentrations who had received 13 CAB/RPV injections over approximately two years before treatment interruption, both above commonly used pharmacokinetic target thresholds. The second case involved a 60-year-old woman with advanced HIV-1 infection and metastatic anal cancer who discontinued treatment after three injections. Five months later, HIV-1 RNA remained undetectable despite very low plasma CAB/RPV concentrations, both below commonly used pharmacokinetic target thresholds. The literature reports another similar case of sustained virologic control 18 months after LA CAB/RPV discontinuation despite inadequate adherence on oral antiretroviral treatment. Altogether, these cases underscore the need for further research to better understand the pharmacological and viro-immunological mechanisms underlying this phenomenon.

Long-acting (LA) injectable formulations of cabotegravir (CAB) and rilpivirine (RPV) are among the guideline-recommended options for the maintenance treatment of virologically suppressed people with HIV [1, 2]. Their use offers clear advantages in terms of adherence and quality of life by eliminating the need for daily oral antiretroviral therapy (ART). However, a major concern associated with LA CAB/RPV is the prolonged pharmacokinetic “tail” following treatment discontinuation, during which drug concentrations progressively decline over months. The pharmacokinetic tail refers to the prolonged period during which measurable drug concentrations persist after the last injection because of slow release from the intramuscular depot. This tail is considered clinically problematic because it may expose patients to drug concentrations below commonly used pharmacokinetic target thresholds that are insufficient to maintain viral suppression while still exerting selective pressure, potentially leading to virologic rebound and resistance [3]. Long-acting CAB/RPV concentrations can persist for up to 12 months or longer after discontinuation, but the clinically used pharmacokinetic target is usually the 4×protein-adjusted inhibitory concentration 90% (PA-IC90) threshold, not simple detectability [3]. Therapeutic drug monitoring studies commonly use target concentrations of 664 ng/mL for CAB (corresponding to 4× PA-IC90) and 32 ng/mL for RPV (upper limit of the first quartile of trough concentrations), although these thresholds were not specifically developed to predict virologic outcomes after treatment discontinuation [4].

Clinical trials and real-world cohorts have consistently shown that virologic suppression is maintained as long as injections are administered according to the recommended schedule, typically every 4 or 8 weeks [5–8]. Conversely, prolonged interruptions have generally been associated with viral rebound, reinforcing the recommendation to promptly resume an alternative fully suppressive ART regimen when LA CAB/RPV is discontinued [7, 8].

To date, only one case report has described sustained virologic suppression for more than one year after discontinuation of LA CAB/RPV without documented antiretroviral treatment exposure during most of the follow-up period. That report highlighted the complexity of interpreting pharmacokinetic and virologic data for LA antiretroviral agents and raised questions about interindividual variability. However, data remain extremely limited, and virologic outcomes after prolonged discontinuation of injectable ART are unknown [9].

Here, we report two additional real-world cases of patients who remained virologically suppressed for several months after discontinuation of LA CAB/RPV without alternative antiretroviral treatment coverage. Virological suppression was maintained despite different clinical contexts and pharmacokinetic profiles. Thus, these cases challenge the prevailing assumption that virologic rebound is inevitable beyond the pharmacokinetic tail and underscore important gaps in our understanding of LA injectable ART.

## METHODS

We identified two patients followed at the HIV Unit of the Geneva University Hospitals who received LA CAB and RPV injections administered every eight weeks and who subsequently experienced prolonged interruptions of treatment without virologic rebound. Clinical, virological, and pharmacological data were extracted from medical records and available stored samples. Plasma HIV-1 RNA levels were measured using standard assays, with virologic suppression defined as HIV-1 RNA <50 copies/mL. Plasma concentrations of CAB and RPV were measured using a validated multiplex liquid chromatography-tandem mass spectrometry (LC-MS/MS) assay allowing simultaneous quantification of several antiretroviral drugs, including CAB and RPV [10]. The lower limits of quantification were 25 ng/mL for CAB and 5 ng/mL for RPV. The same assay was used to screen for the presence of alternative antiretroviral agents in plasma. The absence of alternative ART was also assessed through detailed clinical interviews, review of pharmacy dispensing records and broad antiretroviral screening in plasma. for both cases, analysis of insurance reimbursement records for Case 1, communication with the general practitioner involved in the care of Case 2. No alternative antiretroviral agent was detected in either patient. HIV genotypic resistance testing was not performed in the absence of virologic failure and replication, and proviral DNA genotyping in the absence of viremia has limited interpretability for resistance selection. Both cases were anonymized, and patients had provided consent for use of their clinical data.

## CASE 1

**Figure 1.**
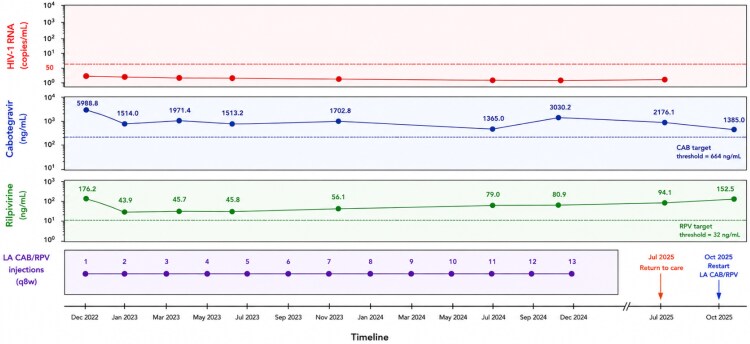
Longitudinal virologic and pharmacokinetic profile of Case 1. The first panel shows plasma HIV-1 RNA levels (copies/mL). The horizontal dashed line indicates the threshold for virologic suppression (50 copies/mL). The second and third panels display plasma cabotegravir (CAB) and rilpivirine (RPV) concentrations, respectively, on a logarithmic scale. Horizontal dashed lines indicate pharmacokinetic target thresholds: 664 ng/mL for CAB (corresponding to 4× the protein-adjusted 90% inhibitory concentration [PA-IC90]) and 32 ng/mL for RPV (corresponding to the upper limit of the first quartile of trough concentrations [Q1 Ctrough]). The last panel illustrates treatment exposure over time. The patient received 13 LA CAB/RPV injections administered every 8 wk, with the last injection in December 2024, followed by treatment interruption, return to care in July 2025, and resumption of LA CAB/RPV in October 2025. Abbreviations: CAB, cabotegravir; RPV, rilpivirine; LA, long-acting; PA-IC90, protein-adjusted 90% inhibitory concentration; Q1 Ctrough, first quartile of trough plasma concentrations.

A 54-year-old man with HIV infection diagnosed in 2002, documented virological suppression since 2008 under efavirenz/emtricitabine/tenofovir disoproxil fumarate and then with RPV/emtricitabine/tenofovir alafenamide from 2019 until end of 2022. At HIV diagnosis, HIV-1 RNA was approximately 34 000 copies/mL and the CD4 count was 226 cells/mm^3^. In December 2022, he was switched to intramuscular injections of LA CAB/RPV every eight weeks with excellent tolerance and sustained virologic suppression. He received a total of 13 injections between December 2022 and December 2024, when the last injection was administered. Subsequently, he missed several scheduled visits due to psychosocial factors, including work-related stress and failure to receive appointment reminders. He returned to care in July 2025, approximately seven months after his last injection. Surprisingly, plasma HIV-1 RNA remained undetectable. Plasma CAB and RPV concentrations were 1385 ng/mL and 79 ng/mL, respectively, both remaining above commonly used pharmacokinetic target thresholds despite the prolonged interruption (Figure 1). No oral antiretroviral intake was reported. Broad antiretroviral screening performed by LC-MS/MS did not detect any alternative antiretroviral agent in plasma. No concomitant medication known to significantly affect CAB or RPV metabolism was identified. There was no intercurrent illness that could explain the observed pharmacokinetic profile. After obtaining consent from the patient, we asked the health insurance to provide all medication reimbursement records for the preceding year to verify that the patient had not received oral or injectable ART elsewhere. Given the unexpected virologic control, the patient was temporarily started on oral darunavir/cobicistat/emtricitabine/tenofovir alafenamide for one month while his virologic history was clarified. He subsequently resumed LA CAB/RPV injections without complications. At the time of manuscript submission, he remained virologically suppressed following reinitiation of LA CAB/RPV in October 2025.

## CASE 2

**Figure 2.**
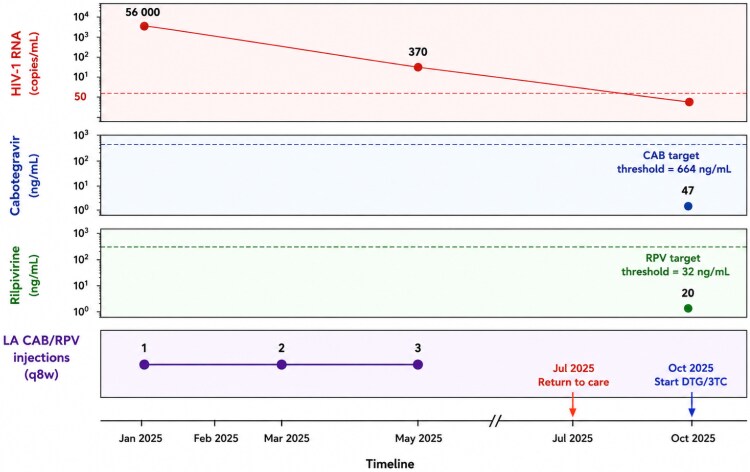
Longitudinal virologic and pharmacokinetic profile of Case 2. The first panel shows plasma HIV-1 RNA over time with the threshold for virologic suppression (50 copies/mL). The second and third panels display plasma cabotegravir and rilpivirine concentrations measured in October 2025. Horizontal dashed lines represent target drug concentration thresholds: 664 ng/mL for CAB (corresponding to 4× protein-adjusted 90% inhibitory concentration [PA-IC90]) and 32 ng/mL for RPV (upper limit of the first quartile of trough concentrations [Q1 Ctrough]). The last panel illustrates treatment exposure over time, with markers indicating injection time points and treatment interruption thereafter. Abbreviations: CAB, cabotegravir; RPV, rilpivirine; LA, long-acting; PA-IC90, protein-adjusted 90% inhibitory concentration; Q1 Ctrough, first quartile of trough plasma concentrations.

A 60-year-old woman presented in January 2025 with advanced HIV disease and metastatic anal cancer. She had been diagnosed with HIV infection in 1987 and had interrupted ART between 2018 and 2024 in the context of HIV denial and intolerance to multiple oral antiretroviral regimens. At HIV diagnosis, HIV-1 RNA was approximately 140 000 copies/mL. A historical genotypic resistance test performed in 2003 did not identify any resistance-associated mutations. In January 2025, the patient presented with HIV-1 RNA of 56 000 copies/mL and a CD4 count of 35 cells/mm^3^ (8%). No opportunistic infection was identified, but metastatic anal cancer was diagnosed. Given the lack of tolerated oral antiretroviral options and the urgency of achieving viral suppression, LA CAB/RPV was initiated in February 2025 despite detectable viremia, following multidisciplinary discussion and with the patient's agreement. She received three injections, with the last administered in May 2025. Shortly thereafter, she elected to discontinue all antiretroviral and oncologic treatments. No oral ART was taken subsequently. The general practitioner responsible for home follow-up confirmed that no antiretroviral medication was taken during this period. Broad antiretroviral screening performed by LC-MS/MS did not detect any alternative antiretroviral agent in plasma. In October 2025, five months after stopping injections, she was hospitalized for cancer-related pain. Unexpectedly, plasma HIV-1 RNA remained <50 copies/mL, with a CD4 count of 52 cells/mm^3^ (9%). Plasma CAB and RPV concentrations were 47 ng/mL and 20 ng/mL, respectively, both below commonly used pharmacokinetic target thresholds (Figure 2). She started oral dolutegravir/lamivudine, which was well tolerated and maintained virologic suppression. She died one month later from progression of her metastatic cancer.

## DISCUSSION

We describe two people with HIV who remained virologically suppressed for several months after discontinuation of LA CAB/RPV without alternative ART coverage. These cases occurred in markedly different clinical contexts and were associated with distinct pharmacokinetic profiles. In the first case, viral suppression persisted for seven months after the last injection, with plasma concentrations of both drugs remaining above commonly used pharmacokinetic target thresholds. The second patient maintained virologic suppression five months after treatment discontinuation despite very low plasma concentrations for both CAB and RPV.

Clinical trials such as ATLAS, FLAIR, and ATLAS-2 M, as well as real-world cohorts including CARES and RELATIVITY, have demonstrated the efficacy of LA CAB/RPV when administered according to protocol [5–7, 11, 12]. In these studies, virologic rebound was uncommon during on-schedule treatment butincreased substantially with prolonged interruptions. Pharmacokinetic studies indicate that CAB and RPV exhibit prolonged elimination phases after the last injection, resulting in a pharmacokinetic tail that may persist for several months. However, drug concentrations progressively decline over time and eventually fall below commonly used pharmacokinetic target thresholds [13]. This has led to strong recommendations to initiate alternative suppressive ART promptly after discontinuation.

Only one published case has described sustained virologic suppression 13–18 months after discontinuation of LA CAB/RPV [9]. In that report, CAB concentrations were below the limit of quantification, while RPV was 40.5 ng/mL (ie, 3.4 times above the protein-adjusted IC90) 18 months postadministration. A non-nucleoside reverse transcriptase inhibitor resistance mutation was detected on genotypic testing performed during virologic suppression. Our two cases add to the limited literature describing prolonged virologic suppression after discontinuation of LA CAB/RPV and highlight substantial heterogeneity in pharmacokinetic and virologic outcomes. Compared with the single previously published case, our report includes two patients with distinct clinical contexts and divergent drug concentration profiles, strengthening the plausibility of a true biological phenomenon rather than an isolated anomaly. One possible explanation for the first case is prolonged drug persistence resulting from slower elimination and/or prolonged release from the intramuscular depot. Cabotegravir is primarily metabolized by UGT1A1, with a minor contribution from UGT1A9, while RPV is mainly metabolized by CYP3A4 [3, 14]. Although pharmacogenetic testing was not performed, interindividual variability in these pathways may contribute to prolonged drug exposure. The second case exhibited low plasma CAB/RPV concentrations despite maintained virologic suppression. One possible explanation is that plasma concentrations do not fully reflect total drug exposure after LA injection. Experimental models have shown that macrophages accumulate around the injection depot and internalize CAB particles, creating a secondary intracellular reservoir that may contribute to prolonged drug persistence beyond what is reflected by plasma concentrations [15]. In addition, variability in tissue distribution, local blood flow, and depot release may contribute to differences between plasma and tissue drug exposure [16]. The hypothesis of a transient post-treatment control cannot be excluded, however, such hypothesis remains uncertain in the context of advanced HIV infection, no immune reconstitution and terminal malignancy, conditions generally not associated with effective immune-mediated viral control. These considerations remain hypothetical, particularly for Case 2, as only a single postdiscontinuation pharmacokinetic measurement was available. These contrasting observations further highlight the complexity of interpreting virologic outcomes after discontinuation of LA injectable therapy. In the reported cases, resistance testing was not performed in the context of virologic suppression. While proviral DNA genotyping can sometimes be considered in this context, its clinical interpretation is limited because detected mutations may represent archived variants within the viral reservoir rather than resistance selected under ongoing viral replication [17, 18].

More broadly, these findings highlight the complexity of LA injectable antiretroviral pharmacology. Injectable formulations involve processes including drug depot formation in muscle tissue, gradual systemic release, intracellular uptake, and variable tissue distribution. While oral ART pharmacokinetics are relatively predictable and reversible, injectable formulations involve complex processes including drug depot formation in muscle tissue, gradual systemic release, and variable tissue distribution [16, 19]. Interindividual variability in muscle composition, local blood flow, metabolism, and tissue distribution may all contribute to prolonged antiviral activity in some individuals. At present, these mechanisms remain incompletely understood. Importantly, our findings do not challenge current recommendations regarding the management of LA CAB/RPV interruptions. Prolonged discontinuation remains associated with a significant risk of virologic rebound, and initiation of alternative suppressive therapy remains the standard of care. However, the small number of cases reported in the literature, including the present series, suggests that individual responses to treatment discontinuation may vary considerably. Additional pharmacokinetic, virologic, and immunologic studies are therefore needed to better understand the determinants of prolonged viral suppression after interruption of LA injectable ART.
